# P-658. Cost-Effectiveness of V116, an Adult Specific 21-Valent Pneumococcal Conjugate Vaccine, vs. PCV20 on Pneumococcal Disease in Japan--A Delta-Price Approach

**DOI:** 10.1093/ofid/ofae631.855

**Published:** 2025-01-29

**Authors:** Peter P Mueller, Atsushi Tajima, Taizo Matsuki, Kelsie Cassell, Nicole Cossrow, Zinan Yi, Kelly D Johnson, Kwame Owusu-Edusei

**Affiliations:** Merck, Boston, Massachusetts; MSD K.K., Chiyoda, Tokyo, Japan; MSD K.K., Chiyoda, Tokyo, Japan; Merck, Boston, Massachusetts; Merck & Co, Inc., Kenilworth, New Jersey; Merck, Boston, Massachusetts; Merck & Co., Inc, North Wales, PA; Merck & Co., Inc., Rahway, New Jersey

## Abstract

**Background:**

V116 is an investigational 21-valent pneumococcal conjugate vaccine (PCV) specifically designed for adults. It includes eight unique serotypes (15A, 15C [generated from deOAc-15B], 16F, 23A, 23B, 24F, 31 and 35B) not covered by any currently licensed pneumococcal vaccine (Figure 1). This study used the delta-price approach to conduct a cost-effectiveness analysis (CEA) of vaccinating adults aged 65+ years with V116 vs. PCV20 in Japan.

**Figure:**
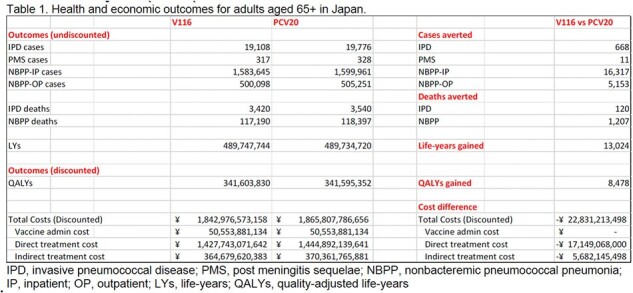

**Methods:**

A Markov model was built to track the lifetime health and economic outcomes of using V116 compared to PCV20 among adults in Japan. The same vaccine effectiveness values were assumed for the two vaccines. We assumed a vaccination coverage rate of 39% based on current Japanese reports of vaccination rates in 65-year-olds. Costs were adjusted to 2024 JPY and a 2% discount rate for both costs and quality-adjusted life-years (QALYs) were used to perform the CEA. The CEA was conducted from societal (direct and indirect costs included) and health care (only direct costs) perspectives. A premium price (price difference vs. PCV20) was used in the analysis. The summary results were presented as incremental cost-effectiveness ratios (ICERs).

**Figure:**
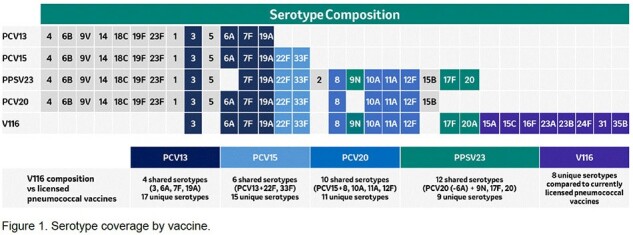

**Results:**

Vaccinating 39% of adults aged 65+ years with V116 prevented approximately 113,000 total cases of pneumococcal disease (PD) and 6,700 disease-related deaths; meanwhile PCV20 prevented approximately 91,000 total cases of PD and 5,400 disease-related deaths (Table 1). When compared to PCV20, V116 yielded an additional 8,500 QALYs, reduced medical costs by 17.15 billion JPY, and reduced indirect costs by 5.68 billion JPY.

V116 was cost-saving up to a premium price of 1,213 JPY and 1,615 JPY from the health care and societal perspectives, respectively. At an ICER of 5 million JPY/QALY gained, V116 was cost-effective up to a premium price of 4,210 JPY and 4,612 JPY from the health care and societal perspectives, respectively (Figure 2). Model outcomes were most sensitive to discount rates and medical costs for NBPP (Figure 3).

**Figure:**
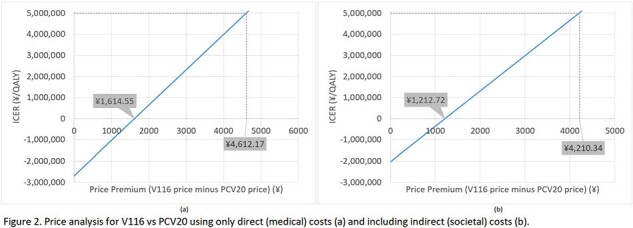

**Conclusion:**

At a premium price between 4,210 and 4,612 JPY, V116 remained cost-effective when compared to PCV20 in Japanese adults 65+ years; and a premium price between 1,213 and 1,615 JPY was found to be cost-saving for the same strategy comparison.

**Figure:**
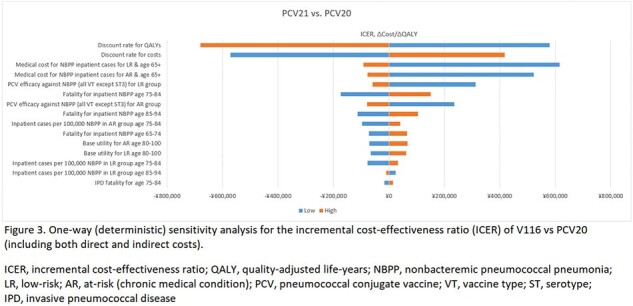

**Disclosures:**

**Peter P. Mueller, PhD**, Merck & Co. Inc.: Stocks/Bonds (Public Company) **Atsushi Tajima, n/a**, Merck & Co. Inc.: Stocks/Bonds (Public Company)|MSD: Employee **Taizo Matsuki, n/a**, GSK: Past employee by October 2023|MSD: Study and publication fund|MSD: Stocks/Bonds (Public Company) **Nicole Cossrow, PhD**, Merck: Stocks/Bonds (Public Company) **Zinan Yi, n/a**, Merck: Stocks/Bonds (Public Company) **Kelly D. Johnson, PhD**, Merck & Co., Inc.: Employee|Merck & Co., Inc.: Stocks/Bonds (Public Company) **Kwame Owusu-Edusei, Ph.D.**, Merck & Co., Inc., Rahway, NJ, USA: Employee|Merck & Co., Inc., Rahway, NJ, USA: Stocks/Bonds (Public Company)

